# DEMENTIA CARE AND BEHAVIORAL HEALTH WORKFORCE DEVELOPMENT: INNOVATIONS IN TRAINING ACROSS SETTINGS

**DOI:** 10.1093/geroni/igz038.1389

**Published:** 2019-11-08

**Authors:** Mary F Wyman, Verena R Cimarolli, Robyn Stone

**Affiliations:** 1 WS MIDDLETON MEMORIAL VETERANS HOSPITAL, Madison, Wisconsin, United States; 2 LeadingAge LTSS Center @UMass Boston, Washington, District of Columbia, United States; 3 LeadingAge, Washington, District of Columbia, United States

## Abstract

It is well-established that there is a critical shortage of trained health care staff who can deliver high-quality behavioral health services to persons with dementia. The development of innovative professional training curricula, and effectively implementing and sustaining such programs, is a key element in addressing this workforce crisis. This symposium highlights cutting-edge efforts being conducted across the continuum of care, to train health care professionals to support optimal behavioral health for persons with dementia. In the outpatient setting, Wyman et al. present data from a survey of mental health clinicians on their perspectives about working with persons with dementia and caregivers, revealing critical gaps in knowledge and skills to target in continuing education programming. Wray and colleagues focus on integrated behavioral health in primary care, discussing how geriatric experts can most effectively contribute to improved assessment and treatment within this setting. Koepp presents an innovative program to transform outpatient mental health care for persons with dementia through provider training and consultation. In the residential care setting, Reinhardt and colleagues report on the implementation of a comprehensive approach to the alleviation of behavioral health problems through training direct care staff in person-directed care in nursing homes. Finally, Karel et al share how VA interprofessional nursing home teams learn and sustain an evidence-based program to address behavioral concerns among residents with dementia. The discussant will lend a deep expertise in research and policy related to the geriatric workforce to her remarks on the presentations.

